# Association Between Women’s Birth Weight and Reproductive Characteristics in Adulthood: The JPHC-NEXT Study

**DOI:** 10.2188/jea.JE20240305

**Published:** 2025-10-05

**Authors:** Shiori Itoi, Chie Nagata, Aurelie Piedvache, Naho Morisaki, Kohei Ogawa, Yoshiko Yamamoto, Isao Saito, Koutatsu Maruyama, Kazuhiko Arima, Kiyoshi Aoyagi, Kozo Tanno, Kazumasa Yamagishi, Isao Muraki, Nobufumi Yasuda, Rieko Kanehara, Taiki Yamaji, Motoki Iwasaki, Manami Inoue, Shoichiro Tsugane, Norie Sawada

**Affiliations:** 1 Department of Social Medicine, National Center for Child Health and Development, Tokyo, Japan; 2 Department of Obstetrics and Gynecology, The University of Tokyo, Tokyo, Japan; 3 Department of Obstetrics and Gynecology, The Jikei University School of Medicine, Tokyo, Japan; 4 Department of Health Promotion, Integrated Center for Women’s Health, National Center for Child Health and Development, Tokyo, Japan; 5 Center for Maternal-Fetal, Neonatal and Reproductive Medicine, National Center for Child Health and Development, Tokyo, Japan; 6 Department of Health Policy, National Center for Child Health and Development, Tokyo, Japan; 7 Department of Public Health and Epidemiology, Faculty of Medicine, Oita University, Oita, Japan; 8 Department of Bioscience, Graduate School of Agriculture, Ehime University, Ehime, Japan; 9 Department of Public Health, Nagasaki University Graduate School of Biomedical Sciences, Nagasaki, Japan; 10 Department of Hygiene and Preventive Medicine, Iwate Medical University, Iwate, Japan; 11 Department of Public Health, Graduate School of Medicine, Juntendo University, Tokyo, Japan; 12 Department of Public Health Medicine, Institute of Medicine, and Health Services Research and Development Center, University of Tsukuba, Tsukuba, Japan; 13 Ibaraki Western Medical Center, Ibaraki, Japan; 14 Public Health, Department of Social Medicine, Graduate School of Medicine, Osaka University, Osaka, Japan; 15 Department of Public Health, Kochi University Medical School, Kochi, Japan; 16 Division of Cohort Research, National Cancer Center Institute for Cancer Control, Tokyo, Japan; 17 Division of Epidemiology, National Cancer Center Institute for Cancer Control, Tokyo, Japan; 18 Division of Prevention, National Cancer Center Institute for Cancer Control, Tokyo, Japan; 19 International University of Health and Welfare Graduate School of Public Health, Tokyo, Japan

**Keywords:** birth weight, menarche, menopause, menstrual irregularity, reproductive characteristics

## Abstract

**Background:**

We aimed to investigate the association between women’s birth weight and their reproductive characteristics.

**Methods:**

We used data from the Japan Public Health Center-based Prospective Study for the Next Generation (JPHC-NEXT), a population-based cohort in Japan. The main analysis included 40,796 women aged 40 to 68 years. Outcomes of interest were age at menarche, age at menopause, history of menstrual irregularity, and nulliparity. Associations between self-reported birth weight categories and outcomes were assessed using either a linear regression or a modified Poisson regression adjusted for potential confounders.

**Results:**

Among participants with complete data, those with lower birth weights (<1,500 g and 1,500–2,499 g) compared to women with a birth weight of 3,000–3,999 g had a later age at menarche (adjusted mean difference [aMD]: 2.4 months and 2.0 months, respectively), earlier age at menopause (aMD: −6.7 months and −2.7 months, respectively), and therefore a shorter reproductive span (aMD: −7.7 months and −4.5 months, respectively). They also had a higher risk of menstrual irregularity (adjusted relative risk [aRR]: 1.19 and 1.11, respectively) and a higher likelihood of nulliparity (aRR: 1.25 and 1.19, respectively).

**Conclusion:**

We observed that Japanese women’s birth weight was significantly associated with reproductive characteristics. Specifically, those with a low birth weight had a shorter reproductive span and a higher risk of irregular menses and nulliparity compared to those with a normal birth weight.

## INTRODUCTION

In recent years, numerous studies have shown how early life *in utero* and childhood can influence subsequent well-being and longevity in adulthood. The largest body of evidence exists for increased cardiovascular and non-communicable disease risks associated with those with low birth weight^1^ and subsequent catch-up growth.^2^^,^^3^ Now widely known as the developmental origins of health and adult disease (DOHaD),^4^ the association between babies born small at birth and increased risks of diseases, especially cardiovascular and metabolic diseases, has been consistently studied.

Recent studies have suggested that early life environments may also influence reproductive systems. Low birth weight or small for gestational age (SGA) is commonly used as an indicator of conditions *in utero*, with previous studies showing those born with low birth weight or SGA are associated with various outcomes, including earlier onset of puberty and menarche,^5^^–^^8^ reduced uterine and ovarian size,^9^^,^^10^ ovarian hypo-responsiveness to follicular stimulating hormone,^11^^,^^12^ and increased risk of ovulation disorders.^13^

Although these studies may be suggestive of an association between birth weight and reproductive characteristics, studies assessing birth weight and fertility have been conflicting. Studies using a cohort of pregnant women reported that SGA is not associated with infertility compared to those born appropriate for gestational age (AGA), with no differences in time to pregnancy.^14^ Similarly, studies examining those with infertility diagnosis have found no increased risk of infertility among those in the SGA group compared to those with AGA.^15^ This disparity may be because studies have utilized specific populations, such as individuals seeking fertility treatment or those amid their reproductive span (typically in their 20s to 30s), rather than targeting individuals who have completed their reproductive span regardless of pregnancy experience.

However, some larger studies utilizing nationwide cohort datasets have suggested the adverse effects on fertility of low birthweight or SGA. An analysis of the Danish national cohort study targeting pregnant women reported a longer time to pregnancy for low maternal birthweight.^16^ Similarly, a Swedish cohort study reported from a study population in their 20s that a reduced probability of giving birth for those born preterm or of very low birth weight was observed, although the SGA group seemed more likely to have given birth compared to those born AGA.^17^ A subsequent study in 2006, utilizing an updated dataset from Ekholm’s study, found that the increase in reproduction rate for SGA seen in the previous report was less clear when the population reached their 30s.^18^

Currently, studies focusing on those who have finished their reproductive span are lacking. Furthermore, studies on birthweight and reproductive characteristics have been conducted in predominantly Caucasian populations, highlighting the need for more diverse studies. In Japan, the proportion of low birth weight has increased in the last three decades.^19^ Therefore, a better understanding of the impact of women’s birth weight on their reproductive characteristics in this population is needed. We aimed to conduct a study assessing the relationship between low birth weight and subsequent reproductive health for those with completed reproductive years, utilizing a Japanese nationwide cohort study.

## METHODS

### Study population

**Figure 1.**
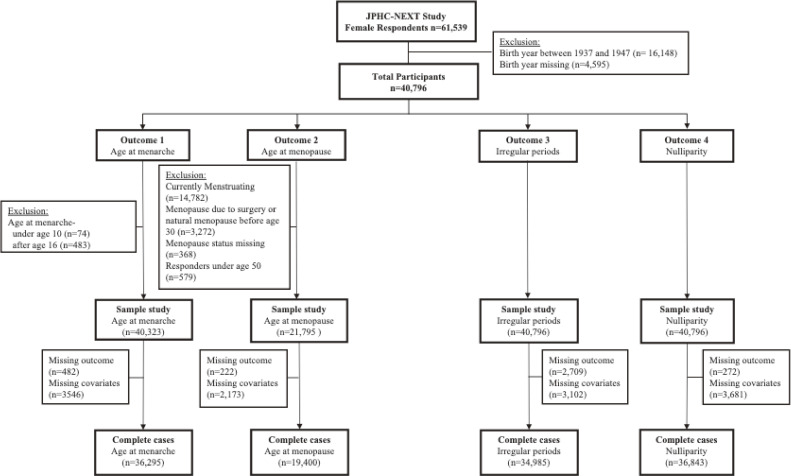
Flow chart of the study population

The Japan Public Health Center-based Prospective Study for the Next Generation (JPHC-NEXT) is a population-based cohort study conducted in 16 municipalities of seven prefectures across Japan, initiated in 2011. The initial aim of this large cohort study was to elucidate the risk factors for lifestyle-related diseases and contribute to the development of personalized healthcare.^20^ Residents aged 40–74 years were invited to participate in the study, resulting in a cohort of 52,566 males and 61,539 females. Participants filled out a self-administered questionnaire, which included details of their birth weight, medical history, and lifestyle information. Data from female participants collected between 2011 and 2016 were used in this study. Restriction to those who were born in or after 1948 and answered their birth weight resulted in 40,796 women (Figure 1). While the dataset contains respondents born between 1937 and 1977, we removed those born before 1948 because the birth weight was missing in more than 30% of subjects each year before 1948. Moreover, this date corresponds to the year when the Maternal and Child Health Handbook, a personal document that monitors pregnancy and childhood health issued by the local government to all pregnant women, was disseminated in Japan. As a result, birth weight data is more likely to be more accurate after this year. Detailed descriptions of the full study design and protocol are described elsewhere.^20^^,^^21^

### Ethics approval

The JPHC-NEXT study protocol was approved by the Institutional Review Board at the National Cancer Center (No. 2011-186) as well as at each collaborating institution of the study areas. The analysis protocol for this study was approved by the Institutional Review Board at the National Cancer Center (No. 2017-250) as well as the National Center for Child Health and Development on June 4, 2018 (No. 1847).

### Measurements

The exposure variable of interest in this study was women’s self-reported birth weight. In the questionnaire, participants were asked to choose one of the following categories classifying their weight at birth: <1,500 g, 1,500–2,499 g, 2,500–2,999 g, 3,000–3,999 g, >4,000 g, and “do not know”. Those with missing data on birth weight were excluded.

The outcomes of interest were age at menarche, age at menopause, reproductive span, menstrual irregularity, and nulliparity. Age at menarche was assessed from the questionnaire response, “At what age did you start menses?” Responses under 10 years old and over 16 years old (±2 standard deviation) were excluded as they may be input errors or caused by medical conditions or traumatic events. Menstrual irregularity was assessed through the question, “Are your periods regular? If you have reached menopause, please answer according to the premenopausal state.” Nulliparity was assessed from the questionnaire response to “Have you ever given birth?” Age at menopause was assessed from two-step questions, the first of which was “Do you still have periods?” A negative answer then required a distinction between natural and iatrogenic causes and asked for the age at menopause. We selected respondents of natural menopause, with a current age between 50 and 69 years, and an age at menopause above 30 years. Age 50 years was chosen as the cut-off to ensure that respondents have reached menopause, rather than being in a transient amenorrheic state. Reproductive span was defined as the interval between the age at menarche and the age at natural menopause. Figure 1 summarizes the exclusion and inclusion criteria for each outcome.

Other covariates chosen to be included in this study were based on past literature. Covariates were as follows: birth year, place of residence, having an older sibling, passive smoking around 10 years old (almost none, 1–3 times a month, 1–4 times a week, almost every day), body mass index (BMI) at age 20 years, current height categorized into quartiles, smoking status (never, current, former), current marital status, and educational attainment (junior high school, high school, junior college/technical school/dropout from college, college degree or above, and missing). The place of residence was included to account for variability in the recruitment population and regional differences in both birth weight and subsequent fertility outcomes. We adjusted for place of residence as a fixed effect to control for these regional variations. Place of residence was categorized based on study sites, which included Yokote (Akita), Saku (Nagano), Ninohe/Karumai (Iwate), Unzen/Minamishimabara (Nagasaki), Ozu (Ehime), Konan/Aki (Kochi), and Chikusei (Ibaraki). BMI at 20 years old was estimated by dividing the weight at 20 years old in kilograms by the current height in meters and categorized into three groups: <18.5, 18.5–24.9, and ≥25.0 kg/m^2^.

### Statistical analysis

The participants’ characteristics and outcomes were expressed for each birth weight category, and missing birth weight, in percentages for categorical data and mean and standard deviation for continuous data. Multivariate analyses on complete cases were performed to investigate the association between birth weight and outcomes. A complete case was defined as no missing data for the covariates previously mentioned. Mean differences (MD) and their 95% confidence intervals (CIs), expressed in months, of age at menarche and age at menopause were estimated with a linear regression model. For irregular periods and nulliparity, relative risks (RR) and their 95% CIs were estimated with a generalized linear model with a Poisson distribution and robust standard errors.

The basic model (model 1) was adjusted for birth year and place of residence. Additional adjustments were made for socio-demographic factors that may widely affect an individual’s reproductive characteristics (reported current height, having an older sibling, and passive smoking at age 10) (model 2). For age at menarche, to assess the influence of catch-up growth and adiposity at adolescence, adjusting for the BMI at 20 years, a proxy for the pubertal growth, in addition to factors used in model 2, was performed (model 3a). For the analysis of age at menopause and nulliparity, in addition to factors used in model 2, adjustments were performed to account for factors that may influence reproduction, including smoking status, educational attainment, BMI at 20 years old, and marital status (model 3b). For the analysis of irregular periods, in addition to factors used in model 2, further adjustments were performed to account for factors that may influence reproductive function, including smoking status, educational attainment, and BMI at 20 years old (model 3c).

Several sensitivity analyses were additionally conducted. First, in our dataset, the missingness of outcomes and covariates ranged from 10% to 14%. For this reason, a sensitivity analysis was conducted on women who had given information about their birth weight, but incomplete for others; we created twenty imputed datasets by chained equations using Rubin’s combination rules (eTable 1).^22^ Next, for age at menopause and nulliparity, models further adjusting for the other reproductive outcomes that precede the outcome of interest (ie, age at menarche, and irregular periods for nulliparity; age at menarche, irregular periods and nulliparity for menopause), was also conducted, as previous studies have suggested strong correlation between these outcomes (model 4a–c). Models further including a combination of these terms were also considered (model 5a–b). Third, to assess whether our results were subject to a disproportion of chances to have a child by marital status or to recall bias for those who had undergone menopause, we conducted a sub-group analysis on nulliparity limiting to those who were married, as well as a sub-group analysis on menstrual irregularity stratified by menopausal status. Finally, given the secular trend in birthweight, we further examined whether the effect of birthweight was modified by birth year categories. We categorized birth years quadrennially, except for the first category (2 years) and the last category (7 years). This was included as a categorical variable in the models to avoid linear assumptions. For this sensitivity analysis, we first re-ran all models with an interaction term between birth year categories and birthweight. We then presented the change/risk estimates stratified by birth year categories.

Data were analyzed using Stata Statistical Software Release 18 (StataCorp, College Station, TX, USA).

## RESULTS

**Figure 2.**
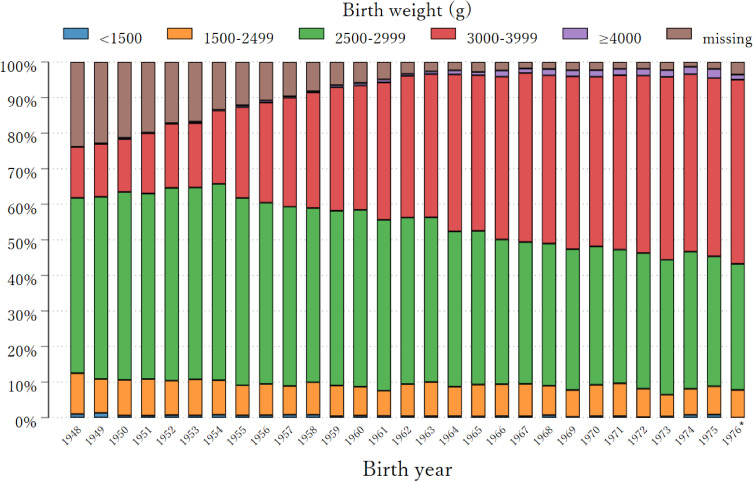
Distribution of birthweight of the study population by birth year (1948–1977) ^*^Only 4 cases were born in 1977; 1976 and 1977 were so combined to avoid a misinterpretation of birth weight distribution in 1977.

The exclusion and inclusion criteria for each outcome, shown in Figure 1, resulted in participants aged 40 to 68 years and an average age of 54 years. Participants’ characteristics by each category of birth weight are shown in Table 1. On average, the age at menarche was 13.2 (standard deviation [SD], 1.3) years. Those with a birth weight of 3,000–3,999 g had an average age at menarche of 12.9 (SD, 1.3) years, while those with a birth weight of <1,500 g had a later average onset of menarche at ages 13.0 (SD, 1.4) years. On average, women had their menopause at age 50.7 (SD, 3.2) years. Those with a birth weight of 3,000–3,999 g had an average onset of menopause at 50.7 (SD, 3.0) years, while those with a birth weight of <1,500 g and 1,500–2,999 g had an earlier average onset of menopause at ages 50.2 (SD, 4.1) years and 50.5 (SD, 3.5) years, respectively. About 18% and 8.5% of women reported irregular periods and nulliparity, respectively, with a higher proportion amongst those with a birth weight <1,500 g (20.8% and 15.7%, respectively). In this study, women with low birthweight were more likely to exhibit a lower BMI at 20 years old, a shorter adult height, and lower educational attainment compared to those with higher birthweight categories (nonparametric tests for trend *P*-value <0.001 for all three covariates). Women with lower birth weight were also more likely to be born between 1948 and 1959. The distribution of birth weight categories across different birth years is shown in Figure 2. Predominant birth weight categories were 2,500–2,999 g for years 1948–1967 and then predominantly 3,000–3,999 g for later years.

**Table 1.** Participant characteristics

	Total	Women with missing birth weight data		Women with birth weight data	Birth weight									

					<1,500 g		1,500–2,499 g		2,500–2,999 g		3,000–3,999 g		≥4,000 g	
**Total**	45,391	4,595	(10.1)	40,796	293	(0.7)	4,095	(10.0)	21,602	(53.0)	14,453	(35.4)	353	(0.9)

**Birth year**														
1948–1949	5,098	1,190	(25.9)	3,908	60	(20.5)	534	(13.0)	2,565	(11.9)	744	(5.1)	5	(1.4)
1950–1954	11,003	1,964	(42.7)	9,039	74	(25.3)	1,095	(26.7)	5,902	(27.3)	1,937	(13.4)	31	(8.8)
1955–1959	9,162	876	(19.1)	8,286	64	(21.8)	788	(19.2)	4,625	(21.4)	2,764	(19.1)	45	(12.7)
1960–1964	7,614	296	(6.4)	7,318	39	(13.3)	637	(15.6)	3,577	(16.6)	3,003	(20.8)	62	(17.6)
1965–1969	6,623	150	(3.3)	6,473	29	(9.9)	567	(13.8)	2,694	(12.5)	3,087	(21.4)	96	(27.2)
1970–1977	5,891	119	(2.6)	5,772	27	(9.2)	474	(11.6)	2,239	(10.4)	2,918	(20.2)	114	(32.3)

**Age at menarche** ^a^														
Reported, *n*	44,205	4,364		39,841	283		3,970		21,090		14,157		341	
Mean (SD), years	12.8 (1.3)	13.2	(1.3)	12.8 (1.3)	13.0	(1.4)	12.9	(1.3)	12.9	(1.3)	12.7	(1.3)	12.5	(1.3)
Missing, *n*	629	147	(3.3)	482	5	(1.7)	58	(1.4)	248	(1.2)	166	(1.2)	5	(1.5)
**Age at menopause** ^a^														
Reported, *n*	25,081	3,508		21,573	193		2,354		12,976		5,961		89	
Mean (SD), years	50.7 (3.2)	50.6	(3.5)	50.7 (3.2)	50.2	(4.1)	50.5	(3.5)	50.7	(3.2)	50.7	(3.0)	50.7	(3.5)
Missing, *n*	292	70	(2.0)	222	2.0	(1.0)	31	(1.3)	135	(1.0)	54	(0.9)	0	(0.0)

**Irregular periods**														
Reported “No”	33,506	2,906	(63.2)	30,600	193	(65.9)	2,924	(71.4)	16,066	(74.4)	11,141	(77.1)	276	(78.2)
Reported “Yes”	8,349	862	(18.8)	7,487	61	(20.8)	814	(19.9)	3,919	(18.1)	2,631	(18.2)	62	(17.6)
Missing	3,536	827	(18.0)	2,709	39	(13.3)	357	(8.7)	1,617	(7.5)	681	(4.7)	15	(4.2)
**Nulliparity**														
Reported “No”	40,250	4,114	(89.5)	36,136	243	(82.9)	3,537	(86.4)	19,401	(89.8)	12,661	(87.6)	294	(83.3)
Reported “Yes”	4,777	389	(8.5)	4,388	46	(15.7)	519	(12.7)	2,069	(9.6)	1,699	(11.8)	55	(15.6)
Missing	364	92	(2.0)	272	4	(1.4)	39	(1.0)	132	(0.6)	93	(0.6)	4	(1.1)

**Height at baseline survey, cm**														
<151	7,970	1,304	(28.4)	6,666	105	(35.8)	1,171	(28.6)	4,204	(19.5)	1,174	(8.1)	12	(3.4)
151–155	13,303	1,515	(33.0)	11,788	102	(34.8)	1,310	(32.0)	6,924	(32.1)	3,406	(23.6)	46	(13.0)
156–159	11,976	1,007	(21.9)	10,969	50	(17.1)	921	(22.5)	5,675	(26.3)	4,222	(29.2)	101	(28.6)
≥160	11,921	660	(14.4)	11,261	34	(11.6)	673	(16.4)	4,738	(21.9)	5,622	(38.9)	194	(55.0)
Missing, *n* (%)	221	109	(2.4)	112	2	(0.7)	20	(0.5)	61	(0.3)	29	(0.2)	0	(0.0)

**BMI at age 20 years,** kg/m^2^														
<18.5	6,305	424	(9.2)	5,881	56	(19.1)	770	(18.8)	3,131	(14.5)	1,884	(13.0)	40	(11.3)
18.5–24.9	35,008	3,157	(68.7)	31,851	209	(71.3)	3,013	(73.6)	17,009	(78.7)	11,347	(78.5)	273	(77.3)
≥25.0	2,137	223	(4.9)	1,914	17	(5.8)	197	(4.8)	908	(4.2)	771	(5.3)	21	(5.9)
Missing	1,941	791	(17.2)	1,150	11	(3.8)	115	(2.8)	554	(2.6)	451	(3.1)	19	(5.4)

**Smoking status**														
Never smoked	36,178	3,829	(83.3)	32,349	223	(76.1)	3,205	(78.3)	17,510	(81.1)	11,149	(77.1)	262	(74.2)
Current smoker	4,293	328	(7.1)	3,965	34	(11.6)	433	(10.6)	1,944	(9.0)	1,510	(10.4)	44	(12.5)
Past smoker	4,601	351	(7.6)	4,250	31	(10.6)	428	(10.5)	2,027	(9.4)	1,719	(11.9)	45	(12.7)
Missing	319	87	(1.9)	232	5	(1.7)	29	(0.7)	121	(0.6)	75	(0.5)	2	(0.6)

**Passive smoking around 10 years old**														
Almost none	20,529	2,017	(43.9)	18,512	121	(41.3)	1,809	(44.2)	10,031	(46.4)	6,401	(44.3)	150	(42.5)
1–3 times a month	1,874	116	(2.5)	1,758	6	(2.0)	173	(4.2)	924	(4.3)	632	(4.4)	23	(6.5)
1–4 times a week	3,754	249	(5.4)	3,505	26	(8.9)	292	(7.1)	1,856	(8.6)	1,303	(9.0)	28	(7.9)
Almost every day	16,375	1,448	(31.5)	14,927	106	(36.2)	1,564	(38.2)	7,573	(35.1)	5,543	(38.4)	141	(39.9)
Missing	2,859	765	(16.6)	2,094	34	(11.6)	257	(6.3)	1,218	(5.6)	574	(4.0)	11	(3.1)

**Marital status**														
Single	2,528	160	(3.5)	2,368	24	(8.2)	280	(6.8)	1,042	(4.8)	991	(6.9)	31	(8.8)
Married/divorced/partner	42,429	4,223	(91.9)	38,206	264	(90.1)	3,786	(92.5)	20,437	(94.6)	13,398	(92.7)	321	(90.9)
Missing	434	212	(4.6)	222	5	(1.7)	29	(0.7)	123	(0.6)	64	(0.4)	1	(0.3)

**Educational attainment**														
Junior high school	5,249	1,374	(29.9)	3,875	65	(22.2)	574	(14.0)	2,507	(11.6)	713	(4.9)	16	(4.5)
High school	23,419	2,204	(48.0)	21,215	151	(51.5)	2,143	(52.3)	11,517	(53.3)	7,243	(50.1)	161	(45.6)
Junior college/specialty/college ​ or university dropout	13,157	733	(16.0)	12,424	59	(20.1)	1,098	(26.8)	6,061	(28.1)	5,072	(35.1)	134	(38.0)
College/university/graduate ​ school	2,838	62	(1.3)	2,776	11	(3.8)	216	(5.3)	1,215	(5.6)	1,295	(9.0)	39	(11.0)
Missing	728	222	(4.8)	506	7	(2.4)	64	(1.6)	302	(1.4)	130	(0.9)	3	(0.8)

**Having older siblings**														
None	17,459	1,322	(28.8)	16,137	111	(37.9)	1,766	(43.1)	8,418	(39.0)	5,714	(39.5)	128	(36.3)
One	14,256	1,198	(26.1)	13,058	88	(30.0)	1,174	(28.7)	6,563	(30.4)	5,090	(35.2)	143	(40.5)
Two or more	13,676	2,075	(45.2)	11,601	94	(32.1)	1,155	(28.2)	6,621	(30.6)	3,649	(25.2)	82	(23.2)

Data are shown as *n* (%), unless otherwise noted.

^a^Sample sizes differ due to differing exclusion criteria, which is shown in detail in Figure 1.

Table 2 shows the difference (in months) from the reference birth weight category (3,000–3,999 g) for age at menarche and menopause, and relative risks for irregular periods and nulliparity, adjusting for possible confounders. For age at menarche, those with birth weight of <1,500 g and 1,500–2,499 g had a later age at menarche (model 3a: adjusted mean difference [aMD] 2.4 months; 95% CI, 0.4–4.3 months, aMD 2.0 months; 95% CI, 1.5–2.6 months, respectively). For age at menopause, those with birth weight of <1,500 g and 1,500–2,499 g had an earlier age at menopause (model 3b: aMD −6.7 months; 95% CI, −12.7 to −0.7 months, aMD −2.7 months; 95% CI, −4.6 to −0.7 months, respectively). Therefore, the overall reproductive span was shorter for those with a birth weight of <1,500 g and 1,500–2,999 g, compared to those with a birth weight of 3,000–3,999 g (model 3b: aMD −7.7 months; 95% CI, −14.1 to −1.2 months, aMD −4.5 months; 95% CI, −6.6 to −2.4 months, respectively) (results not shown). Risk for irregular periods was higher for individuals born at 1,500–2,499 g, but a difference was not observed for those born <1,500 g (model 3c: adjusted relative risk [aRR] 1.11; 95% CI, 1.03–1.20, aRR 1.19; 95% CI, 0.94–1.51, respectively). Similarly, for nulliparity, a higher risk was observed for those born at 1,500–2,499 g, but a significant difference was not observed for those born <1,500 g (model 3b: aRR 1.19; 95% CI, 1.11–1.29, aRR: 1.25; 95% CI, 0.99–1.58, respectively).

**Table 2.** Adjusted mean difference (MD) and relative risk (RR) for each outcome

	Birth weight				

	<1,500 g	1,500–2,499 g	2,500–2,999 g	3,000–3,999 g	≥4,000 g
**Age at menarche, MD [95% CI] (*N* = 36,295** ^a^ **)**					
Total, *n* (%)	236 (0.7)	3,564 (9.8)	19,144 (52.8)	13,040 (35.9)	311 (0.9)
Model 1^b^	2.1 [0.1–4.0]	1.8 [1.2–2.3]	0.9 [0.6–1.2]	ref	0.1 [−1.6 to 1.8]
Model 2^c^	2.9 [0.9–4.8]	2.4 [1.8–3.0]	1.3 [0.9–1.6]	ref	−0.3 [−2.0 to 1.4]
Model 3a^d^	2.4 [0.4–4.3]	2.0 [1.5–2.6]	1.1 [0.7–1.4]	ref	−0.2 [−1.8 to 1.5]
**Age at menopause, MD [95% CI] (*N* = 19,400** ^a^ **)**					
Total, *n* (%)	154 (0.8)	2,070 (10.7)	11,671 (60.2)	5,422 (28.0)	83 (0.4)
Model 1^b^	−9.0 [−15.0 to −3.0]	−4.0 [−5.9 to −2.1]	−1.8 [−3.0 to −0.6]	ref	0.8 [−7.3 to 8.9]
Model 2^c^	−8.2 [−14.2 to −2.2]	−3.3 [−5.3 to −1.4]	−1.4 [−2.6 to −0.2]	ref	0.5 [−7.6 to 8.6]
Model 3b^e^	−6.7 [−12.7 to −0.7]	−2.7 [−4.6 to −0.7]	−1.1 [−2.3 to 0.2]	ref	1.1 [−7.0 to 9.2]

**Irregular periods, RR [95% CI] (*N* = 34,985** ^a^ **)**					
Total, *n* (%)	220 (0.6)	3,407 (9.7)	18,311 (52.4)	12,739 (36.4)	308 (0.9)
Model 1^b^	1.28 [1.01–1.62]	1.16 [1.08–1.25]	1.02 [0.98–1.07]	ref	0.94 [0.74–1.21]
Model 2^c^	1.24 [0.98–1.57]	1.14 [1.06–1.23]	1.02 [0.97–1.07]	ref	0.95 [0.74–1.21]
Model 3c^f^	1.19 [0.94–1.51]	1.11 [1.03–1.20]	1.00 [0.96–1.05]	ref	0.95 [0.74–1.22]
**Nulliparity, RR [95% CI] (*N* = 36,843** ^a^ **)**					
Total, *n* (%)	241 (0.7)	3,640 (9.9)	19,434 (52.8)	13,211 (35.9)	317 (0.9)
Model 1^b^	1.57 [1.16–2.12]	1.36 [1.23–1.49]	1.02 [0.96–1.09]	ref	1.21 [0.94–1.56]
Model 2^c^	1.59 [1.17–2.15]	1.37 [1.25–1.51]	1.04 [0.97–1.11]	ref	1.19 [0.92–1.53]
Model 3b^e^	1.25 [0.99–1.58]	1.19 [1.11–1.29]	1.07 [1.02–1.12]	ref	1.09 [0.90–1.31]

CI, confidence interval; MD, mean difference; RR, relative risk.

^a^Sample sizes differ due to differing exclusion criteria, which is shown in detail in Figure 1.

^b^Model 1 is adjusted for birth year and place of residence for all outcomes.

^c^Model 2 is adjusted for model 1 plus passive smoking at 10 years old, height and having an older brother or sister for all outcomes.

^d^Model 3a is adjusted for model 2 plus body mass index at 20 years old for age at menarche.

^e^Model 3b is adjusted for model 2 plus smoking status, educational attainment, body mass index at 20 years old and marital status.

^f^Model 3c is adjusted for model 2 plus smoking status, educational attainment, body mass index at 20 years old.

Imputing covariates and outcomes when missing did not impact estimates (eTable 2), except for the relative risk of nulliparity, which was higher after imputation for those born with a birth weight of <1,500 g (after imputation, model 3b: aRR 1.32; 95% CI, 1.07–1.63 vs before imputation: aRR 1.25; 95% CI, 0.99–1.58). For age at menopause and nulliparity, further adjustments for preceding reproductive characteristics did not impact estimates (models 4a–5b). After the exclusion of unmarried women (*n* = 2,082, 6%), the relative risk of nulliparity for those born with very low birth weight increased to 1.60 (95% CI, 0.99–2.60) (model 3b), and after imputation to 1.77 (95% CI, 1.17–2.68) (model 3b) (eTable 3). The association between birthweight and risk of irregular periods was similar between women who had undergone menopause and those who were still menstruating (results not shown).

**Figure 3.**
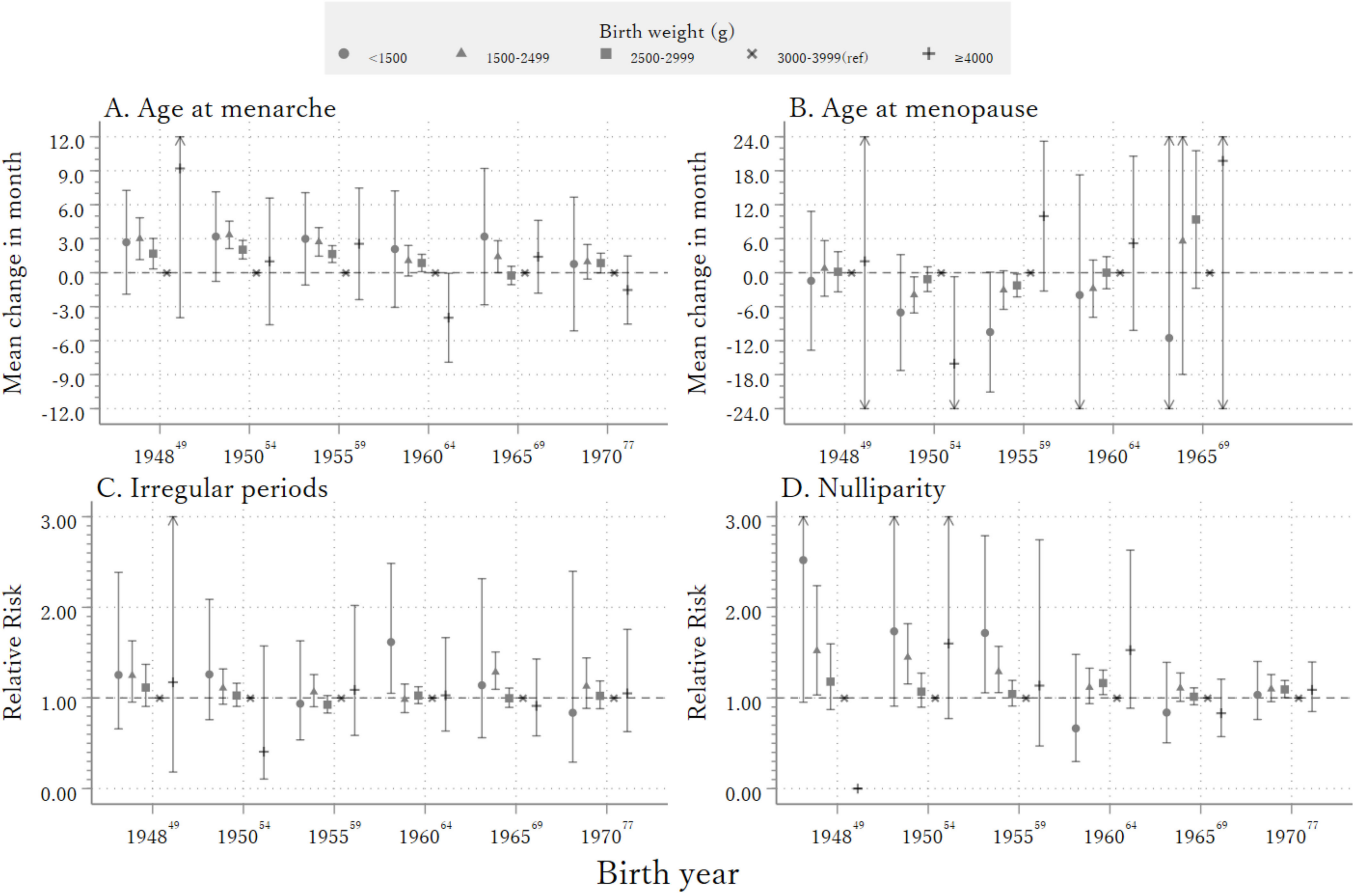
Association between birthweight and age at menarche (**A**), age at menopause (**B**), irregular periods (**C**), and nulliparity (**D**) by birth year (A) model is adjusted for place of residence, passive smoking at 10 years old, height, having an older brother or sister, and body mass index at 20 years old; (B) model is adjusted for place of residence, passive smoking at 10 years old, height, having an older brother or sister, educational attainment, current smoking, body mass index at 20 years old and marital status; (C) model is adjusted for place of residence, passive smoking at 10 years old, height, having an older brother or sister, educational attainment, current smoking and body mass index at 20 years old (D) model is adjusted for place of residence, passive smoking at 10 years old, height, having an older brother or sister, educational attainment, current smoking, body mass index at 20 years old and marital status.

**Figure 4.**
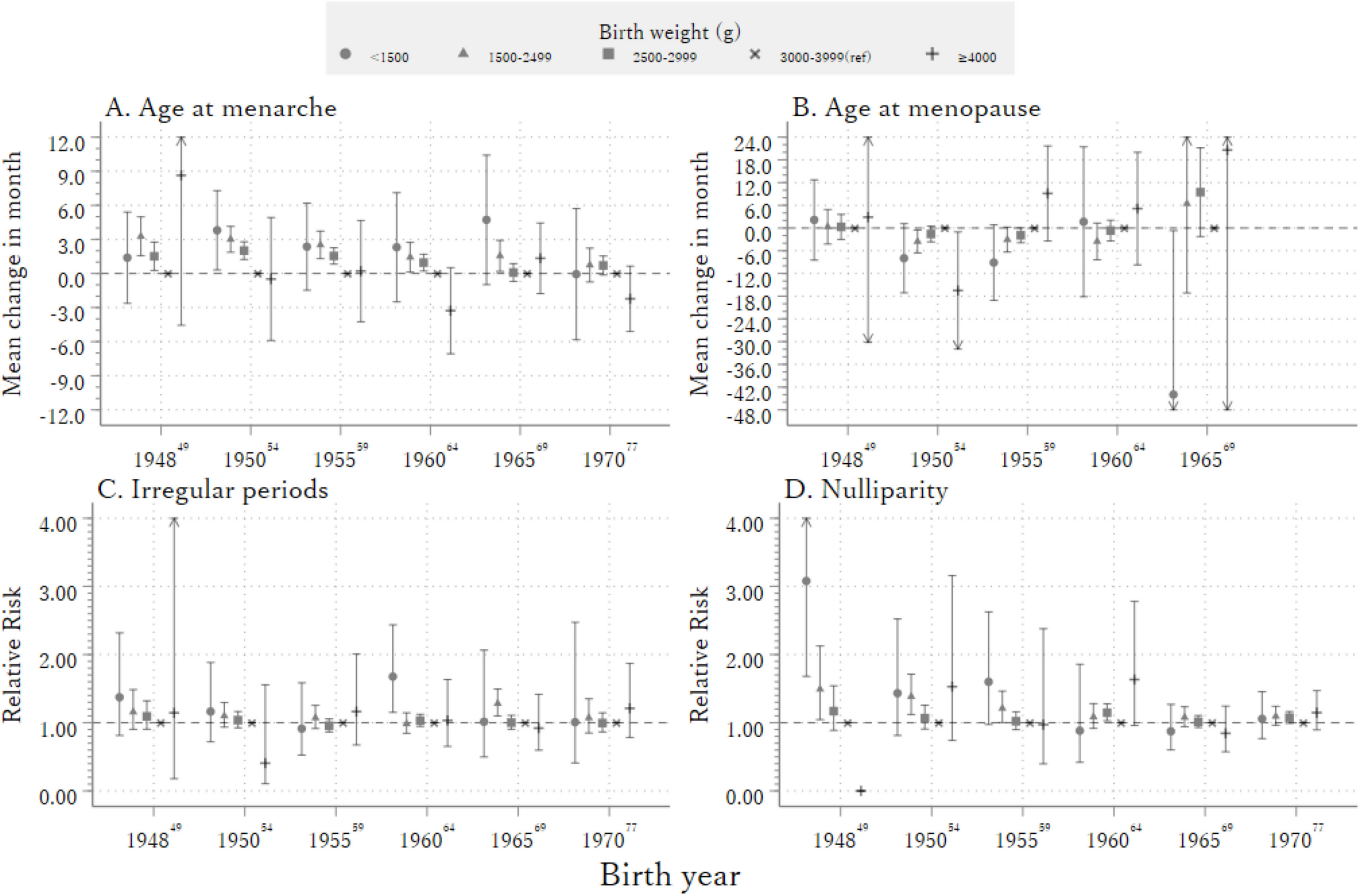
Association between birthweight and age at menarche (**A**), age at menopause (**B**), irregular periods (**C**), and nulliparity (**D**) by birth year (imputed data) (A) model is adjusted for place of residence, passive smoking at 10 years old, height, having an older brother or sister, and body mass index at 20 years old; (B) model is adjusted for place of residence, passive smoking at 10 years old, height, having an older brother or sister, educational attainment, current smoking, body mass index at 20 years old and marital status; (C) model is adjusted for place of residence, passive smoking at 10 years old, height, having an older brother or sister, educational attainment, current smoking and body mass index at 20 years old (D) model is adjusted for place of residence, passive smoking at 10 years old, height, having an older brother or sister, educational attainment, current smoking, body mass index at 20 years old and marital status.

Lastly, we investigated the impact of birth year on the association between birth weight and outcomes (Figure 3A, Figure 3B, Figure 3C, and Figure 3D). Overall *P*-values for the interaction term between the effect of birth weight and birth year were 0.03, 0.48, 0.61, and <0.001 for age of menarche, age at menopause, menstrual irregularity, and nulliparity, respectively. Women with lower birth weight categories had a later age at menarche, with an association for 1,500–2,499 g and 2,500–2,999 g in the 1950s, which gradually attenuated in the 1960s and 1970s. Increased risks of nulliparity associated with lower birth weight categories were observed only among those born before 1960. Associations were similar after imputation (Figure 4A, Figure 4B, Figure 4C, and Figure 4D).

## DISCUSSION

In our large population-based study in Japan, for those born in the years 1948–1977, we found that compared to those with birth weights of 3,000–3,999 g, those with a lower birth weight (<1,500 g and 1,500–2,499 g) have a later age at menarche of approximately 2 months and an earlier age at menopause of about 3 to 7 months, resulting in a shorter reproductive span of about 5 to 8 months. Furthermore, those with a low birth weight had an increased risk of menstrual irregularity and an increased risk of nulliparity. This association was more pronounced among individuals born in the earlier birth cohorts but diminished in later generations.

To the best of our knowledge, this is the first study to evaluate the association between women’s birth weight and both menarche and menopause concomitantly. Previous studies have investigated the association between birth weight and the onset of either menarche or menopause individually. Studies focusing on birth weight and age at menarche concluded that those born with low birth weight or SGA had their first menstruation early,^6^^,^^7^^,^^22^^–^^26^ some highlighting the vigorous “catch-up growth” during infancy.^26^ A meta-analysis by Deng et al reported that women who presented a small birth weight had 0.3 years earlier menarche.^6^ Regarding the onset of menopause, a systematic review in 2018 found no association between low birth weight and age at natural menopause.^8^ However, Sadrazadeh and colleagues concluded that prenatal and childhood exposure to famine was significantly associated with earlier age at menopause.^8^ Our results agree with this study, finding that those with lower birth weights have an earlier onset of menopause. The observed delay in menarche among those with low birth weight in our study was pronounced in the earlier birth year categories. One possible explanation may be that the earlier birth years were close to the post-war era in Japan, characterized by widespread famine and nutritional deficiency. Another factor could be the large cultural and racial differences from previous studies in Europe/North America. Since Japan became more Westernized in more recent years, earlier cohorts may have experienced different nutritional and environmental conditions. Both possibilities may underscore the importance of “catch-up growth” in the acceleration of menarche for those born with low birth weights. Varying results for birth weight and menarche from previous studies may be due to differences in the postnatal environments. Low birth weight, combined with postnatal undernutrition, may be one of the causes for the association seen in our study.

Regarding irregular periods and nulliparity, higher risks were observed for those born with lower birth weights. These findings align with previous biomedical studies suggesting reproductive function is affected by early life in utero.^27^ Ibanez and colleagues have extensively demonstrated this association, in which SGA girls, compared to AGA girls, were more likely to have altered gonadotropin secretion, impaired ovarian development, and reduced ovarian size with a significant decrease in primordial follicle number.^11^^–^^13^ However, past epidemiological studies on fertility outcomes have been conflicting. Some reported a tendency of lower birth weight to be related to a lower probability of giving birth^17^^,^^18^ and a longer time to pregnancy,^14^ while others reported a null association between SGA and infertility.^14^^,^^15^^,^^28^ It should be noted that these studies had been done on women of reproductive age, and their full reproductive potential was not able to be assessed. Our study’s strength is that our population had an average age of 54, with 55% of women in a post-menopausal state. This allowed us to assess their full reproductive potential, possibly explaining the disparity in results compared to some epidemiological studies.

Our findings have several implications. First, our study suggests that birth weight may be associated with reproductive characteristics, with individuals born with lower birth weight having a higher risk of nulliparity, irregular menses, later menarche, and earlier menopause. In Japan, the high proportion of underweight women and insufficient weight gain during pregnancy, which consequently leads to low birth weight infants, has become a national concern.^19^ The findings from our study underscore the need for intervention and education not only for those born with low birth weight but also for those at risk of giving birth to low birth weight infants, such as those underweight or desiring to be underweight.

This highlights the importance of promoting healthy maternal weight and weight gain during pregnancy, as these efforts could not only reduce the prevalence of low birth weight infants but also prevent their potential long-term reproductive implications in subsequent generations. Public health strategies could incorporate targeted support and educational means to raise awareness about the importance of maternal nutrition and weight management during pregnancy.

Furthermore, for those born with low birth weight, while the outcomes observed in our study have multiple contributing factors and causality is not yet established, previous studies have linked early or late menarche and early menopause to be associated with reduced fecundability.^29^^,^^30^ This underscores the importance of addressing these outcomes, especially early menarche, as part of preconception care, engaging in conversation and education by healthcare providers on future reproductive outcomes, and addressing modifiable lifestyle factors.

The present study has several limitations. First, birth weight, as well as other outcome variables, were self-reported and collected retrospectively. Previous studies suggested that self-reported birth weight should be used with caution due to the increased risk of misclassification and recall bias.^31^^–^^35^ For irregular menses, whether cycles were regular or irregular depended on self-assessment, which may differ from clinical definitions and may also be impacted by the perimenopausal state, in which menstrual irregularity is common. Additionally, the question regarding the history of irregular menses had a higher missing rate (6.6%) compared to questions regarding age at menarche (1.1%) and parity (0.6%). The vast majority (80%) of women who did not answer on irregular menses had gone through natural menopause, suggesting they had a harder time recalling the regularity of their menstrual periods.

Second, we did not collect information on gestational age at birth. Low birth weight can be an outcome of preterm birth and/or fetal growth restriction. Given that their impact on an individual’s health may differ, our study results should be interpreted with caution.

Third, the generalizability of our findings may be limited. Since our study population consisted of an older generation in Japan, and more recent generations have shown slightly different patterns in our study, further studies with younger generations are needed. Participation in our study, particularly the results of those born in earlier decades with low birth weight, may be influenced by survivorship bias. Their nutritional and environmental contexts differ from those of current generations.

Lastly, while our results on reproductive outcomes for those with higher birth weights were insignificant, this may be attributed to the cohort belonging to an older generation of Japanese women, in which high birth weights may be rare. Additionally, a recent study on Japanese women suggests a secular trend of decrease in the age of menarche coinciding with historical events.^36^ Similar trends may also apply to the age of menopause. Studies from Western countries have indicated a potential positive association with the risk of breast cancer, potentially mediated by intrauterine conditions and epigenetic regulation.^37^^,^^38^ Further research is needed to investigate the pathways linking high birth weight, reproductive outcomes, and long-term health outcomes, including breast cancer, while accounting for the secular trends and identifying modifiable factors that could mitigate these risks in Japanese cohorts.

Nonetheless, our study has several strengths. We utilized one of the largest cohort studies in Japan, encompassing multiple generations. Previous research on the association between birth weight and reproductive outcomes has utilized cohorts primarily on young, pregnancy-seeking, or currently pregnant women. However, such cohorts may not fully capture a population’s unbiased reproductive potential, as they do not account for all the births over a woman’s entire reproductive span or capture other reproductive characteristics more eminent later in life. By contrast, our cohort included women aged 40–68 years, with the majority having reached menopausal status at the time of study. This enabled us to assess women’s complete reproductive span and outcomes comprehensively.

To conclude, our study suggests an association between birth weight and reproductive characteristics. Specifically, those born with low birth weight may experience a shorter reproductive span and lower fertility, characterized by later onset of menarche and early menopause, and increased risk of irregular menses and nulliparity. Given the contentious nature of study results and the limitations of our study, further research is needed to elucidate the impact of in-utero conditions on one’s reproductive health in later life.

## SUPPLEMENTARY MATERIAL

eTable 1. Impact of imputation on missing data for covariates and outcomes

eTable 2. Adjusted mean change and adjusted relative risks of outcomes after imputation

eTable 3. Sensitivity analysis, adjusted relative risks of nulliparity among ever-married women
