# Supplementary material for: Association Between Women’s Birth Weight and Reproductive Characteristics in Adulthood: The JPHC-NEXT Study
Source: J Epidemiol. 2025 Oct 5;35(10):432–41. doi: 10.2188/jea.JE20240305 (PMC12420951; doi:10.2188/jea.JE20240305)
Supplement: Supplementary file 1 [file je-35-432-s001.pdf]

eTable 1. Impact of imputation on missing data for covariates and outcomes

	Before imputation										After imputation									
	<1,500 g		1,500–2,499 g		2,500–2,999 g		3,000–3,999 g		≥4,000 g		<1,500 g		1,500–2,499 g		2,500–2,999 g		3,000–3,999 g		≥4,000 g	
**Age at menarche, years, mean (SD)**	13.0	(1.4)	12.9	(1.3)	12.9	(1.3)	12.7	(1.3)	12.5	(1.3)	13.0	(1.4)	12.9	(1.3)	12.9	(1.3)	12.7	(1.3)	12.5	(1.3)
Missing, n	5	(1.7)	58	(1.4)	248	(1.2)	166	(1.2)	5	(1.5)										
**Age at menopause, years, mean (SD)**	50.2	(4.1)	50.5	(3.5)	50.7	(3.2)	50.7	(3.0)	50.7	(3.5)	50.2	(4.1)	50.5	(3.5)	50.7	(3.2)	50.7	(3.0)	50.7	(3.5)
Missing, n	2	(1.0)	31	(1.3)	135	(1.0)	54	(0.9)	0	(0.0)										
**Irregular periods**																				
Reported "No"	193	(65.9)	2,924	(71.4)	16,066	(74.4)	11,141	(77.1)	276	(78.2)	222	(75.8)	3,207	(78.3)	17,362	(80.4)	11,684	(80.8)	287	(81.4)
Reported "Yes"	61	(20.8)	814	(19.9)	3,919	(18.1)	2,631	(18.2)	62	(17.6)	71	(24.2)	888	(21.7)	4,240	(19.6)	2,769	(19.2)	66	(18.6)
Missing	39	(13.3)	357	(8.7)	1,617	(7.5)	681	(4.7)	15	(4.2)										
**Nulliparity**																				
Reported "No"	243	(82.9)	3,537	(86.4)	19,401	(89.8)	12,661	(87.6)	294	(83.3)	246	(84.1)	3,570	(87.2)	19,513	(90.3)	12,739	(88.1)	297	(84.1)
Reported "Yes"	46	(15.7)	519	(12.7)	2,069	(9.6)	1,699	(11.8)	55	(15.6)	47	(15.9)	525	(12.8)	2,089	(9.7)	1,714	(11.9)	56	(15.9)
Missing	4	(1.4)	39	(1.0)	132	(0.6)	93	(0.6)	4	(1.1)										
**Height at baseline survey,** cm																				
<151	105	(35.8)	1,171	(28.6)	4,204	(19.5)	1,174	(8.1)	12	(3.4)	106	(36.2)	1,178	(28.8)	4,218	(19.5)	1,176	(8.1)	12	(3.4)
151–155	102	(34.8)	1,310	(32.0)	6,924	(32.1)	3,406	(23.6)	46	(13.0)	102	(34.9)	1,316	(32.1)	6,942	(32.1)	3,413	(23.6)	46	(13.0)
156–159	50	(17.1)	921	(22.5)	5,675	(26.3)	4,222	(29.2)	101	(28.6)	50	(17.2)	925	(22.6)	5,690	(26.3)	4,231	(29.3)	101	(28.6)
≥160	34	(11.6)	673	(16.4)	4,738	(21.9)	5,622	(38.9)	194	(55.0)	34	(11.7)	676	(16.5)	4,752	(22.0)	5,633	(39.0)	194	(55.0)
Missing, n (%)	2	(0.7)	20	(0.5)	61	(0.3)	29	(0.2)	.	(.)										
**BMI at age 20,** kg/m^2																				
<18.5	56	(19.1)	770	(18.8)	3,131	(14.5)	1,884	(13.0)	40	(11.3)	57	(19.5)	792	(19.3)	3219	(14.9)	1943	(13.4)	41	(11.8)

18.5–24.9	209	(71.3)	3,013	(73.6)	17,009	(78.7)	11,347	(78.5)	273	(77.3)	217	(74.2)	3,099	(75.7)	17445	(80.8)	11703	(81.0)	289	(81.8)
≥25.0	17	(5.8)	197	(4.8)	908	(4.2)	771	(5.3)	21	(5.9)	19	(6.3)	204	(5.0)	938	(4.3)	806	(5.6)	23	(6.4)
Missing	11	(3.8)	115	(2.8)	554	(2.6)	451	(3.1)	19	(5.4)										
**Smoking status**																				
Never smoked	223	(76.1)	3,205	(78.3)	17,510	(81.1)	11,149	(77.1)	262	(74.2)	227	(77.5)	3,228	(78.8)	17,608	(81.5)	11,207	(77.5)	264	(74.7)
Current smoker	34	(11.6)	433	(10.6)	1,944	(9.0)	1,510	(10.4)	44	(12.5)	34	(11.8)	436	(10.6)	1,957	(9.1)	1,519	(10.5)	44	(12.6)
Past smoker	31	(10.6)	428	(10.5)	2,027	(9.4)	1,719	(11.9)	45	(12.7)	31	(10.7)	431	(10.5)	2,037	(9.4)	1,727	(11.9)	45	(12.8)
Missing	5	(1.7)	29	(0.7)	121	(0.6)	75	(0.5)	2	(0.6)										
**Passive smoking around 10 years old**																				
Almost none	121	(41.3)	1,809	(44.2)	10,031	(46.4)	6,401	(44.3)	150	(42.5)	139	(47.4)	1,941	(47.4)	10,683	(49.5)	6,691	(46.3)	155	(43.9)
1–3 times a month	6	(2.0)	173	(4.2)	924	(4.3)	632	(4.4)	23	(6.5)	7	(2.4)	184	(4.5)	978	(4.5)	657	(4.5)	24	(6.7)
1–4 times a week	26	(8.9)	292	(7.1)	1,856	(8.6)	1,303	(9.0)	28	(7.9)	29	(9.8)	310	(7.6)	1,952	(9.0)	1,352	(9.4)	29	(8.2)
Almost every day	106	(36.2)	1,564	(38.2)	7,573	(35.1)	5,543	(38.4)	141	(39.9)	118	(40.4)	1,660	(40.5)	7,989	(37.0)	5,753	(39.8)	145	(41.2)
Missing	34	(11.6)	257	(6.3)	1,218	(5.6)	574	(4.0)	11	(3.1)										
**Marital status**																				
Single	24	(8.2)	280	(6.8)	1,042	(4.8)	991	(6.9)	31	(8.8)	25	(8.6)	282	(6.9)	1,048	(4.9)	994	(6.9)	31	(8.8)
Married/divorced/partner	264	(90.1)	3,786	(92.5)	20,437	(94.6)	13,398	(92.7)	321	(90.9)	268	(91.4)	3813	(93.1)	20,554	(95.1)	13,459	(93.1)	322	(91.2)
Missing	5	(1.7)	29	(0.7)	123	(0.6)	64	(0.4)	1	(0.3)										
**Educational attainment**																				
Junior high school	65	(22.2)	574	(14.0)	2,507	(11.6)	713	(4.9)	16	(4.5)	67	(22.9)	588	(14.4)	2,557	(11.8)	723	(5.0)	16	(4.5)
High school	151	(51.5)	2,143	(52.3)	11,517	(53.3)	7,243	(50.1)	161	(45.6)	155	(52.8)	2,177	(53.2)	11,676	(54.0)	7,312	(50.6)	163	(46.2)
Junior college/specialty/college or university dropout	59	(20.1)	1,098	(26.8)	6,061	(28.1)	5,072	(35.1)	134	(38.0)	60	(20.5)	1,112	(27.2)	6141	(28.4)	5,113	(35.4)	135	(38.2)

College/university/ graduate school	11	(3.8)	216	(5.3)	1,215	(5.6)	1,295	(9.0)	39	(11.0)	11	(3.8)	218	(5.3)	1,229	(5.7)	1,305	(9.0)	39	(11.1)
Missing	7	(2.4)	64	(1.6)	302	(1.4)	130	(0.9)	3	(0.8)										

Data are shown as n (%) unless otherwise noted.

eTable 2. Adjusted mean change and adjusted relative risks of outcomes after imputation

	Birth weight				
	<1,500 g	1,500–2,499 g	2,500–2,999 g	3,000–3,999 g	≥4,000 g
**Age at menarche, MD [95% CI] (N=40,298)**					
Model 1[a]	1.9 [0.1–3.7]	1.8 [1.3–2.3]	0.9 [0.6–1.3]	ref	-0.5 [-2.1 to 1.1]
Model 2[b]	2.7 [0.9–4.4]	2.4 [1.9–3.0]	1.3 [1.0–1.7]	ref	-0.9 [-2.5 to 0.7]
Model 3a[c]	2.3 [0.5–4.0]	2.0 [1.5–2.6]	1.1 [0.8–1.4]	ref	-0.7 [-2.3 to 0.9]
**Age at menopause, MD [95% CI] (N=21,795)**					
Model 1[a]	-8.2 [-13.6 to -2.8]	-4.1 [-5.9 to -2.3]	-2.0 [-3.1 to -0.8]	ref	1.0 [-6.9 to 8.9]
Model 2[b]	-7.2 [-12.7 to -1.8]	-3.4 [-5.2 to -1.5]	-1.5 [-2.7 to -0.3]	ref	0.6 [-7.3 to 8.5]
Model 3b[d]	-5.6 [-11.0 to -0.2]	-2.7 [-4.5 to -0.8]	-1.2 [-2.4 to 0.0]	ref	1.2 [-6.6 to 9.1]
Model 4a[e]	-5.7 [-11.1 to -0.2]	-2.7 [-4.5 to -0.8]	-1.2 [-2.4 to -0.0]	ref	1.2 [-6.6 to 9.1]
Model 4b[f], (N=21,499[g])	-5.1 [-10.5 to 0.3]	-2.3 [-4.2 to -0.4]	-1.1 [-2.3 to 0.1]	ref	0.7 [-7.2 to 8.6]
Model 4c[h]	-5.2 [-10.6 to 0.2]	-2.5 [-4.4 to -0.7]	-1.2 [-2.4 to 0.0]	ref	1.4 [-6.4 to 9.3]
Model 5a[i], (N=21,499[g])	-4.7 [-10.1 to 0.7]	-2.2 [-4.1 to -0.4]	-1.1 [-2.3 to 0.1]	ref	0.9 [-6.9 to 8.8]
**Irregular periods, RR [95% CI] (N=40,796)**					
Model 1[a]	1.29 [1.03–1.62]	1.15 [1.07–1.23]	1.03 [0.99–1.08]	ref	1.00 [0.80–1.25]
Model 2[b]	1.25 [1.00–1.57]	1.13 [1.05–1.21]	1.02 [0.98–1.07]	ref	1.01 [0.80–1.26]
Model 3c[j]	1.20 [0.95–1.50]	1.10 [1.02–1.18]	1.01 [0.97–1.06]	ref	1.01 [0.80–1.26]
**Nulliparity, RR [95% CI] (N=40,796)**					
Model 1[a]	1.73 [1.33–2.25]	1.31 [1.20–1.44]	1.01 [0.95–1.08]	ref	1.15 [0.90–1.47]
Model 2[b]	1.74 [1.33–2.26]	1.32 [1.20–1.44]	1.02 [0.96–1.09]	ref	1.14 [0.89–1.45]
Model 3b[d]	1.32 [1.07–1.63]	1.16 [1.08–1.25]	1.06 [1.01–1.11]	ref	1.12 [0.92–1.35]
Model 4a[e]	1.32 [1.07–1.63]	1.16 [1.08–1.24]	1.06 [1.01–1.11]	ref	1.10 [0.91–1.34]
Model 4b[f], (N=40,298[g])	1.32 [1.07–1.63]	1.16 [1.08–1.25]	1.06 [1.01–1.11]	ref	1.13 [0.93–1.38]
Model 5b[k], (N=40,298[g])	1.32 [1.07–1.63]	1.16 [1.08–1.24]	1.06 [1.02–1.12]	ref	1.12 [0.92–1.36]

CI, confidence interval; MD, mean difference; RR, relative risk.
[a] Model 1 is adjusted for birth year and place of residence for all outcomes

[b] Model 2 is adjusted for model 1 plus passive smoking at 10 years old, height and having an older brother or sister for all outcomes

[c] Model 3a is adjusted for model 2 plus body mass index at 20 years old for age at menarche

[d] Model 3b is adjusted for model 2 plus smoking status, educational attainment, body mass index at 20 years old and marital status

[e] Model 4a is adjusted for model 3 plus irregular periods

[f] Model 4b is adjusted for model 3 plus age at menarche

[g] Sample size is lower because the model includes age at menarche after exclusion of outliers (<10 years and >16 years)
[h] Model 4c is adjusted for model 3 plus nulliparity

[i] Model 5a is adjusted for model 3 plus irregular periods, and age at menarche

[j] Model 3c is adjusted for model 2 plus smoking status, educational attainment, body mass index at 20 years old

[k] Model 5b is adjusted for model 3 plus irregular periods, age at menarche, and nulliparity

eTable 3. Sensitivity analysis, adjusted relative risks of nulliparity among ever-married women

	**Birth weight**				
	<1,500 g	1,500–2,499 g	2,500–2,999 g	3,000–3,999 g	≥4,000 g
Nulliparity, RR [95% CI]<br>*Complete cases (N=34,761)*					
Total, n (%)	222 (0.6)	3,389 (9.8)	18,517 (53.3)	12,346 (35.5)	287 (0.8)
Model 1[a]	1.54 [0.95–2.49]	1.40 [1.21–1.62]	1.09 [0.99–1.21]	ref	1.26 [0.83–1.89]
Model 2[b]	1.60 [0.99–2.59]	1.44 [1.24–1.67]	1.12 [1.02–1.24]	ref	1.23 [0.81–1.84]
Model 3b[c]	1.60 [0.99–2.60]	1.44 [1.24–1.67]	1.14 [1.04–1.26]	ref	1.21 [0.81–1.83]
Nulliparity, RR [95% CI]<br>*Imputed dataset (N=38,428)*					
Model 1[a]	1.81 [1.21–2.72]	1.36 [1.18–1.56]	1.09 [1.00–1.20]	ref	1.31 [0.89–1.91]
Model 2[b]	1.85 [1.23–2.78]	1.38 [1.20–1.59]	1.12 [1.02–1.23]	ref	1.28 [0.87–1.87]
Model 3b[c]	1.77 [1.17–2.68]	1.37 [1.19–1.58]	1.13 [1.03–1.24]	ref	1.28 [0.87–1.88]
Model 4a[d]	1.72 [1.13–2.62]	1.36 [1.18–1.57]	1.13 [1.03–1.24]	ref	1.28 [0.87–1.88]
Model 4b[e], (N=37,970)[f]	1.75 [1.15–2.68]	1.38 [1.19–1.59]	1.14 [1.04–1.25]	ref	1.30 [0.88–1.91]
Model 5b[g], (N=37,970)[f]	1.70 [1.11–2.62]	1.37 [1.19–1.58]	1.14 [1.03–1.25]	ref	1.30 [0.89–1.91]

CI, 95% confidence interval; RR, relative risk.

[a] Model 1 is adjusted for birth year and place of residence for all outcomes

[b] Model 2 is adjusted for model 1 plus passive smoking at 10 years old, height and having an older brother or sister for all outcomes

[c] Model 3b is adjusted for model 2 plus smoking status, educational attainment, body mass index at 20 years old and marital status

[d] Model 4a is adjusted for model 3 plus irregular periods

[e] Model 4b is adjusted for model 3 plus age at menarche

[f] Sample size is lower because the model includes age at menarche after exclusion of outliers (<10 years and >16 years)

[g] Model 5b is adjusted for model 3 plus irregular periods, age at menarche, and nulliparity
